# Aberrant Whole-Brain Functional Connectivity and Intelligence Structure in Children with Primary Nocturnal Enuresis

**DOI:** 10.1371/journal.pone.0051924

**Published:** 2013-01-02

**Authors:** Bing Yu, Hongbin Sun, Hongwei Ma, Miao Peng, Fanxing Kong, Fanxing Meng, Na Liu, Qiyong Guo

**Affiliations:** 1 Department of Radiology, Shengjing Hospital of China Medical University, Shenyang, China; 2 Department of Radiology, 4^th^ Hospital of China Medical University, Shenyang, China; 3 Department of Pediatrics, Shengjing Hospital of China Medical University, Shenyang, China; 4 Psychological Outpatient Service, Shengjing Hospital of China Medical University, Shenyang, China; 5 Department of Radiology, The People’s Hospital of Liaoning Province, Shenyang, China; University of California, San Francisco, United States of America

## Abstract

**Aim:**

To assess the potential relationship between intelligence structure abnormalities and whole-brain functional connectivity in children with primary nocturnal enuresis (PNE) with resting-state functional magnetic resonance imaging (fMRI) to provide insights into the association between these two seemingly unrelated conditions.

**Methods:**

Intelligence testing and fMRI data were obtained from 133 right-handed children, including 67 PNE children (M/F, 39∶28; age, 10.5±1.2 y) and 66 age-matched healthy controls (M/F, 37∶29; age, 10.1±1.1 y). All intelligence tests were performed using the China-Wechsler Intelligence Scale for Children (C-WISC). Each subject’s full intelligence quotient (FIQ), verbal IQ (VIQ), performance IQ (PIQ), and memory/caution (M/C) factor was measured and recorded. Resting state fMRI scans were performed on a 3.0-T MR scanner and post-processed using REST software. Comparisons of z-score correlation coefficients between distinct cerebral regions were used to identify altered functional connectivity in PNE children.

**Results:**

The PNE group had normal FIQ, VIQ, and PIQ values, indicating no significant variation from the control group. However, the M/C factor was significantly lower in the PNE group. Compared to the control group, PNE children exhibited overall lower levels of functional connectivity that were most apparent in the cerebello-thalamo-frontal pathway. The M/C factor significantly correlated with z-scores representing connectivity between Cerebellum_Crus1_L and Frontal_Mid_R.

**Conclusion:**

PNE children exhibit intelligence structure imbalance and attention deficits. Our findings suggest that cerebello-thalamo-frontal circuit abnormalities are likely to be involved in the onset and progression of attention impairment in PNE children.

## Introduction

Primary nocturnal enuresis (PNE) affects up to 20% of young children (<5 years old) and nearly 2% of all young adults. PNE is characterized by involuntary voiding of the bladder during sleep beyond age five, which is the generally accepted age required for complete bladder control development and normal voiding habits during waking hours. PNE can cause significant psychosocial stress, potentially leading to more serious complications later in life.

PNE has been correlated with numerous genetic factors, including deficient arginine vasopressin (AVP) secretion, sleep awareness disorder, and bladder dysfunction. Current research also suggests that behavioral conditions, such as attention deficit hyperactivity disorder (ADHD), increase the risk for persistent PNE in children [1]. Notably, antidiuretic treatments have also been shown to enhance short-term memory; Muller *et al.* administered the antidiuretic hormone analog 1-desamino-8-D-arginine vasopressin (DDAVP) to PNE patients and observed improvements [2]. Similarly, the incidence rate of enuresis in ADHD patients is higher than in healthy children [1], [3], [4].

It is thought that PNE children experience cognitive deficits, which manifest as developmental delay and lower gross intelligence level in comparison with healthy control children [5]. A study by Dai *et al.*, however, indicated that PNE children and age-matched healthy children have similar IQ levels, but PNE children with intelligence structure abnormalities exhibit significantly different memory and attention levels [6].

Current evidence pertaining to the physical mechanism of cognitive deficits in PNE patients remains extremely limited, and the existing study results are not fully consistent. Baeyens *et al*. confirmed that the ratio of prepulse inhibition (PPI) signals, assessed using myoelectric tracing technology while simultaneously monitoring eye-wink reaction stimulation signals, was significantly decreased in PNE patients. This result indicated possible defects in the suppressive function of the brain stem associated with PNE [3]. Conversely, a recent event-related fMRI study performed by Yu *et al.* [7] indicated that PNE children exhibited an increased number of working memory deficits, a condition potentially associated with dysfunction of the left cerebellum,and their voxel-based morphometry (VBM) study revealed reduced gray matter density in the right dorsolateral prefrontal cortex (DLPFC) and left cerebellum of PNE children [8]. In addition, Lei *et al.* [9] reported that response inhibition in children with PNE is associated with a relative delay in maturation of prefrontal cortex circuitry that is known to suppress inappropriate responses, their resting-state fMRI study revealed abnormal spontaneous blood oxygen level-dependent (BOLD) activities in the left inferior frontal gyrus, medial frontal gyrus (posterior cingulate gyrus, middle temporal gyrus, left parietal lobe/inferior parietal lobule), and left midbrain of PNE children [10], and their diffusion tensor imaging study revealed that children with PNE showed both a decrease in fractional anisotropy (FA) and an increase in mean diffusivity (MD) in multiple brain regions, including the frontal lobe, anterior cingulate cortex (ACC), insula, and particularly in the thalamus, compared to healthy children [11].

These studies indicate that multiple brain regions and circuits might be associated with the symptoms of enuresis and cognitive disorders in PNE children. However, the functional connectivity among these brain regions in PNE children was previously unknown.

Intrinsic low-frequency functional correlations measured by fMRI have been successfully applied in mapping brain systems in resting state subjects [12]. Low-frequency (50.08 Hz) fluctuations (LFF) of the BOLD signal in the resting state are considered to be related to spontaneous neuronal activity. These fluctuations have been used to identify functional connectivities between different brain regions, demonstrating that even remotely located regions have functional relationships as indicated by high temporal coherent LFF values [13], [14]. These findings imply the existence of neuronal coordination [15], [16]. In addition, several resting state fMRI studies have shown that LFF correlation patterns were altered in some pathological and behavioral conditions [17], [18]. While fMRI techniques are potentially powerful techniques for mapping abnormal neural circuitry, recognition of functional relationships using fMRI is limited by the technique’s sensitivity to factors that may not be directly involved in anatomical connectivity [19]. Despite these limitations, fMRI is a useful tool for investigating whole-brain functional connectivity with minimal patient discomfort, making the method ideal for assessing altered connectivity in children with PNE.

Whole-brain functional connectivity was explored in order to investigate specific alterations in functional connectivity in children with PNE. Based on previous methods proposed by Tzourio-Mazoyer, the brain was divided into 116 automated anatomical labeling (AAL) regions [20], and correlations between each pair of these regions were analyzed in both PNE and normal control subjects. Significant differences in functional connectivity were determined by comparing the correlation coefficients of each pair of regions between the two groups. We also examined the relationships between altered functional connectivity and intelligence tests.

## Materials and Methods

A total of 147 right-handed children including 75 PNE children (39 male, 36 female; aged 10.4±1.3 y) and 72 healthy control children (40 male, 32 female; aged 10.0±1.2 y) were assessed in the present study. Subjects in each group were matched for age, handedness, and primary school level (grades 4–6).

The study protocol was approved by and performed under the supervision of the ethics committee of the Shengjing Hospital of China Medical University (Shenyang 110004, China, no. 2012PS25K). All participants were informed about the study purposes and protocols, and each participant and their guardian provided written informed consent.

All children in the PNE group met the following inclusion criteria: urination under control during the daytime and involuntary urination during sleep≥ twice a week for more than 6 months and normal blood and urine biochemistry, urine culture, and urine flowmetry. In addition, ultrasound examination of the urinary tract revealed no abnormal kidney or urinary tract defects, residual urine, or other urological or neurological disorders or abnormalities. All included subjects underwent routine MRI examination and had normal results.

Prior to inclusion, all children regularly attended the enuresis outpatient clinic, and no children were included that had previously been treated with any typical or atypical psychoactive drug. Children with a current or historical diagnosis of any neurological and psychiatric diseases according to the Diagnostic and Statistical Manual of Mental Disorders published by the American Psychiatric Association (DSM-IV), especially ADHD, were excluded from the present study.

### Intelligence Evaluation and Data Analysis

Intelligence testing was performed at our hospital using the China-Wechsler Intelligence Scale for Children (C-WISC), revised by Gong *et al.* [21] All children completed 11 individual tests, including 6 speech tests and 5 manipulation tests. The speech tests consisted of tests for Information (I, answering common questions), Comprehension (C, answering the best action under a certain circumstance), Sorting (S, summarize the common aspects of each pair of words in a phrase), Arithmetic (A, solve arithmetic questions with mental calculation), Digit symbol (D, recite numbers in sequential and reverse orders), and Vocabulary (V, sort vocabulary according to the degree of difficulty). The manipulation tests included Picture Completion (PC, point out lack of stroke and the name in the figure), Picture Arrangement (PA, arrange randomly sorted figures in a meaningful story), Block Pattern (BP, decompose cubic blocks into the pattern the experimenters presented), Object Assembly (OA, compose four disassembled pieces into complete figures within a certain time), and Coding (Cd, numbers from 1 to 9 were given a specific mark, and the subjects were required to fill in the blank spaces under each number with the corresponding marks without skipping any).

During intelligence testing, the raw score from each subtest was first transformed into a scaled score, and then the sum of scaled scores-V (I+C+S+A+D+V), the sum of scaled scores-P (PC+PA+BP+OA+Cd), and the total sum of scaled scores were calculated. Finally, the scaled scores were transformed into Verbal intelligence quotient (VIQ), procedure intelligence quotient (PIQ), and full intelligence quotient (FIQ).

The following IQ factors were also calculated accordingly: verbal comprehension factor (VC = I+V+C+S), perceptual organization factor (PO = PC+PA+BP+OA), and memory/caution factor (M/C = A+D+Cd). All intelligence tests were administered by trained professionals who were blinded to the study grouping.

### fMRI Data Acquisition

All studies were performed using a 3.0-T scanner (Intera Achieva; Philips Medical Systems, Best, Netherlands) with an 8-channel Sensitivity Encoding (SENSE) head coil. fMRI scans were performed using gradient echo-planar imaging (EPI) sequences (TR/TE: 2000/30 ms; FOV: 230 mm; matrix: 64×64; layers: 32; slice thickness: 4 mm; scanning plane paralleled with AC-PC line). The duration of the dummy scan was 8 s and was added at the start of each fMRI scan in order to eliminate artifacts induced by chemical shifts and to stabilize the magnetic field. Thus, each fMRI scan was performed over 496 s. In order to stabilize patients during fMRI scanning, a thick ear cushion was applied to fix head placement and to eliminate the effects of constructed defects caused by head movements.

### Statistical Analysis

Data obtained from intelligence testing was analyzed using SPSS 17.0 (IBM, USA) software package. FIQ, VIQ, PIQ, VC, PO, and M/C were expressed as mean±SD. Student’s t-tests or Mann-Whitney U tests were used to compare the two independent groups, depending on if the data were normally or non-normally distributed, respectively. Because we employed Bonferroni corrections for multiple comparisons, only values of *P*<0.0083 were considered statistically significant.

All fMRI data preprocessing was performed using the SPM8 software package (Welcome Department of Cognitive Neurology, London, UK) on the MATLAB (Mathworks, USA) platform. Images were realigned, and differences in slice acquisition time were corrected using temporal realignment to the middle slice. Data were discarded if fMRI head motion was >2 mm or 3°. Images were then resampled to 3 mm × 3 mm × 3 mm voxels in Montreal Neurological Institute (MNI)-labeled space (Bounding Box: [−90 −126 −72; 90 90 108]) and smoothed with an isotropic Gaussian kernel (full width at half maximum: 6 mm). The resultant data were further filtered using a bandpass temporal filter (0.01–0.1 Hz) to reduce the effects of low frequency drift and high frequency physiological noise using the REST software package; nuisance signals such as global mean and head motion parameters were regressed out [22].

Whole-brain functional connectivity was analyzed using the method developed by Liu [19]. The registered fMRI data were segmented into 116 regions using the AAL template described by Tzourio-Mazoyer *et al.* [20], which was used in some previous studies [18], [19], [23]. This parcellation divided the cerebra into 90 regions (45 in each hemisphere) and the cerebella into 26 regions (9 in each cerebellar hemisphere and 8 in the vermis). Table 1 lists their abbreviations and the MNI coordinates of the center of each region.

**Table 1:** Abbreviations and MNI coordinates of AAL.

Index	Abbreviation	MNI coordinate			Index	Abbreviation	MNI coordinate		
		X	Y	Z			X	Y	Z
1	Precentral_L	−39	−6	51	59	Parietal_Sup_L	−23	−60	59
2	Precentral_R	41	−8	52	60	Parietal_Sup_R	26	−59	62
3	Frontal_Sup_L	−18	35	42	61	Parietal_Inf_L	−43	−46	47
4	Frontal_Sup_R	22	31	44	62	Parietal_Inf_R	46	−46	50
5	Frontal_Sup_Orb_L	−17	47	−13	63	SupraMarginal_L	−56	−34	30
6	Frontal_Sup_Orb_R	18	48	−14	64	SupraMarginal_R	58	−32	34
7	Frontal_Mid_L	−33	33	35	65	Angular_L	−44	−61	36
8	Frontal_Mid_R	38	33	34	66	Angular_R	46	−60	39
9	Frontal_Mid_Orb_L	−31	50	−10	67	Precuneus_L	−7	−56	48
10	Frontal_Mid_Orb_R	33	53	−11	68	Precuneus_R	10	−56	44
11	Frontal_Inf_Oper_L	−48	13	19	69	Paracentral_Lobule_L	−8	−25	70
12	Frontal_Inf_Oper_R	50	15	21	70	Paracentral_Lobule_R	7	−32	68
13	Frontal_Inf_Tri_L	−46	30	14	71	Caudate_L	−11	11	9
14	Frontal_Inf_Tri_R	50	30	14	72	Caudate_R	15	12	9
15	Frontal_Inf_Orb_L	−36	31	−12	73	Putamen_L	−24	4	2
16	Frontal_Inf_Orb_R	41	32	−12	74	Putamen_R	28	5	2
17	Rolandic_Oper_L	−47	−8	14	75	Pallidum_L	−18	0	0
18	Rolandic_Oper_R	53	−6	15	76	Pallidum_R	21	0	0
19	Supp_Motor_Area_L	−5	5	61	77	Thalamus_L	−11	−18	8
20	Supp_Motor_Area_R	9	0	62	78	Thalamus_R	13	−18	8
21	Olfactory_L	−8	15	−11	79	Heschl_L	−42	−19	10
22	Olfactory_R	10	16	−11	80	Heschl_R	46	−17	10
23	Frontal_Sup_Medial_L	−5	49	31	81	Temporal_Sup_L	−53	−21	7
24	Frontal_Sup_Medial_R	9	51	30	82	Temporal_Sup_R	58	−22	7
25	Frontal_Med_Orb_L	−5	54	−7	83	Temporal_Pole_Sup_L	−40	15	−20
26	Frontal_Med_Orb_R	8	52	−7	84	Temporal_Pole_Sup_R	48	15	−17
27	Rectus_L	−5	37	−18	85	Temporal_Mid_L	−56	−34	−2
28	Rectus_R	8	36	−18	86	Temporal_Mid_R	57	−37	−1
29	Insula_L	−35	7	3	87	Temporal_Pole_Mid_L	−36	15	−34
30	Insula_R	39	6	2	88	Temporal_Pole_Mid_R	44	15	−32
31	Cingulum_Ant_L	−4	35	14	89	Temporal_Inf_L	−50	−28	−23
32	Cingulum_Ant_R	8	37	16	90	Temporal_Inf_R	54	−31	−22
33	Cingulum_Mid_L	−5	−15	42	91	Cerebellum_Crus1_L	−35	−67	−29
34	Cingulum_Mid_R	8	−9	40	92	Cerebellum_Crus1_R	38	−67	−30
35	Cingulum_Post_L	−5	−43	25	93	Cerebellum_Crus2_L	−28	−73	−38
36	Cingulum_Post_R	7	−42	22	94	Cerebellum_Crus2_R	33	−69	−40
37	Hippocampus_L	−25	−21	−10	95	Cerebellum_3_L	−8	−37	−19
38	Hippocampus_R	29	−20	−10	96	Cerebellum_3_R	13	−34	−19
39	ParaHippocampal_L	−21	−16	−21	97	Cerebellum_4_5_L	−14	−43	−17
40	ParaHippocampal_R	25	−15	−20	98	Cerebellum_4_5_R	18	−43	−18
41	Amygdala_L	−23	−1	−17	99	Cerebellum_6_L	−22	−59	−22
42	Amygdala_R	27	1	−18	100	Cerebellum_6_R	26	−58	−24
43	Calcarine_L	−7	−79	6	101	Cerebellum_7b_L	−31	−60	−45
44	Calcarine_R	16	−73	9	102	Cerebellum_7b_R	34	−63	−48
45	Cuneus_L	−6	−80	27	103	Cerebellum_8_L	−25	−55	−48
46	Cuneus_R	14	−79	28	104	Cerebellum_8_R	26	−56	−49
47	Lingual_L	−15	−68	−5	105	Cerebellum_9_L	−10	−49	−46
48	Lingual_R	16	−67	−4	106	Cerebellum_9_R	10	−49	−46
49	Occipital_Sup_L	−17	−84	28	107	Cerebellum_10_L	−22	−34	−42
50	Occipital_Sup_R	24	−81	31	108	Cerebellum_10_R	27	−34	−41
51	Occipital_Mid_L	−32	−81	16	109	Vermis_1_2	2	−39	−20
52	Occipital_Mid_R	37	−80	19	110	Vermis_3	2	−40	−11
53	Occipital_Inf_L	−36	−78	−8	111	Vermis_4_5	2	−52	−6
54	Occipital_Inf_R	38	−82	−8	112	Vermis_6	2	−67	−15
55	Fusiform_L	−31	−40	−20	113	Vermis_7	2	−72	−25
56	Fusiform_R	34	−39	−20	114	Vermis_8	2	−64	−34
57	Postcentral_L	−42	−23	49	115	Vermis_9	2	−55	−35
58	Postcentral_R	41	−25	53	116	Vermis_10	1	−46	−32

Regional mean time series were estimated by first averaging the time series of all voxels by region. Then, Pearson correlation analysis was conducted for each pair of regions in each subject. After correlation coefficients were computed, Fisher’s r-to-z transformation was applied to the correlation coefficients. Individual z-scores were compared with two-tailed *t*-tests to determine the significance of functional connectivities between the two groups. The false discovery rate (FDR) approach was applied to identify the restriction threshold capable of reducing the proportion of type I errors to <0.05. Pearson correlation analysis was performed between the FIQ, VIQ, PIQ, VC, PO, and M/C of all PNE children, with z-scores representing connectivities with significant differences between the PNE and control groups.

## Results

Based on observed head motion >2 mm or 3° in 8 children of the PNE group and 6 children of the control group, C-WISC and fMRI data were discarded for 14 subjects. The fMRI data of the remaining 67 children in the PNE group (M/F = 39∶28; average age 10.5±1.2 y; 4.87±0.65 y of education) and 66 children in the control group (M/F = 37∶29; 10.1±1.1 y; 4.76±0.68 y of education) were included in the study, resulting in a total of 133 subjects. There were no significant differences in age or years of education (*P* = 0.1275 and *P* = 0.3410, respectively).

### Intelligence Testing

FIQ, VIQ, and PIQ values were normal in the PNE group, showing no significant variation compared with normal controls (*P*>0.0083). The M/C factor, however, was significantly different between the two groups (*P = *0.0060), but no significant difference was observed for VC or PO factors (*P*>0.0083). The intelligence results are shown in detail in Table 2.

**Table 2:** Intelligence testing results (x±s).

	Control group(n = 66)	PNE group(n = 67)	*t* or Z Value(df = 131)	*P* Value
Verbal intelligence quotient (VIQ)	102.70±15.54	96.11±15.09	2.651	0.0090
Procedure intelligence quotient (PIQ)	102.03±15.32	100.70±17.76	−0.603^#^	0.5465
Full intelligence (FIQ)	100.46±13.56	98.57±13.00	0.818	0.415
Verbal comprehension (VC)	104.16±15.35	101.68±14.11	0.971	0.3334
Perceptual organization (PO)	101.69±13.88	102.77±17.49	−0.393	0.6948
Memory/Caution (M/C)	101.43±16.59	89.96±14.52*	4.147	0.0060

* P<0.0083; ^#^Z Value.

### Functional Connectivity Differences

**Figure 1:**
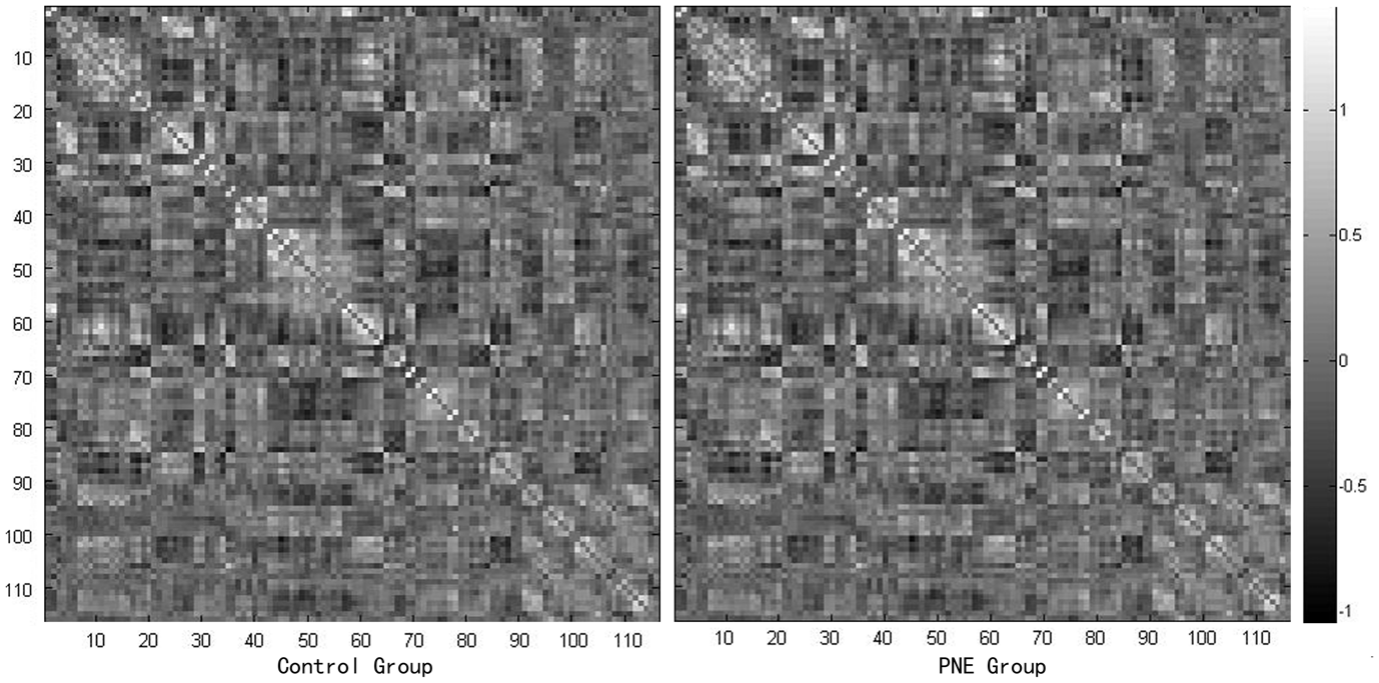
Mean z-score matrices for the control and PNE groups. Each figure shows a 116×116 square matrix in which the x- and y-axes correspond to AAL regions. Each entry indicates the mean z-score representing functional connectivity between each pair of regions in the brain.

**Figure 2:**
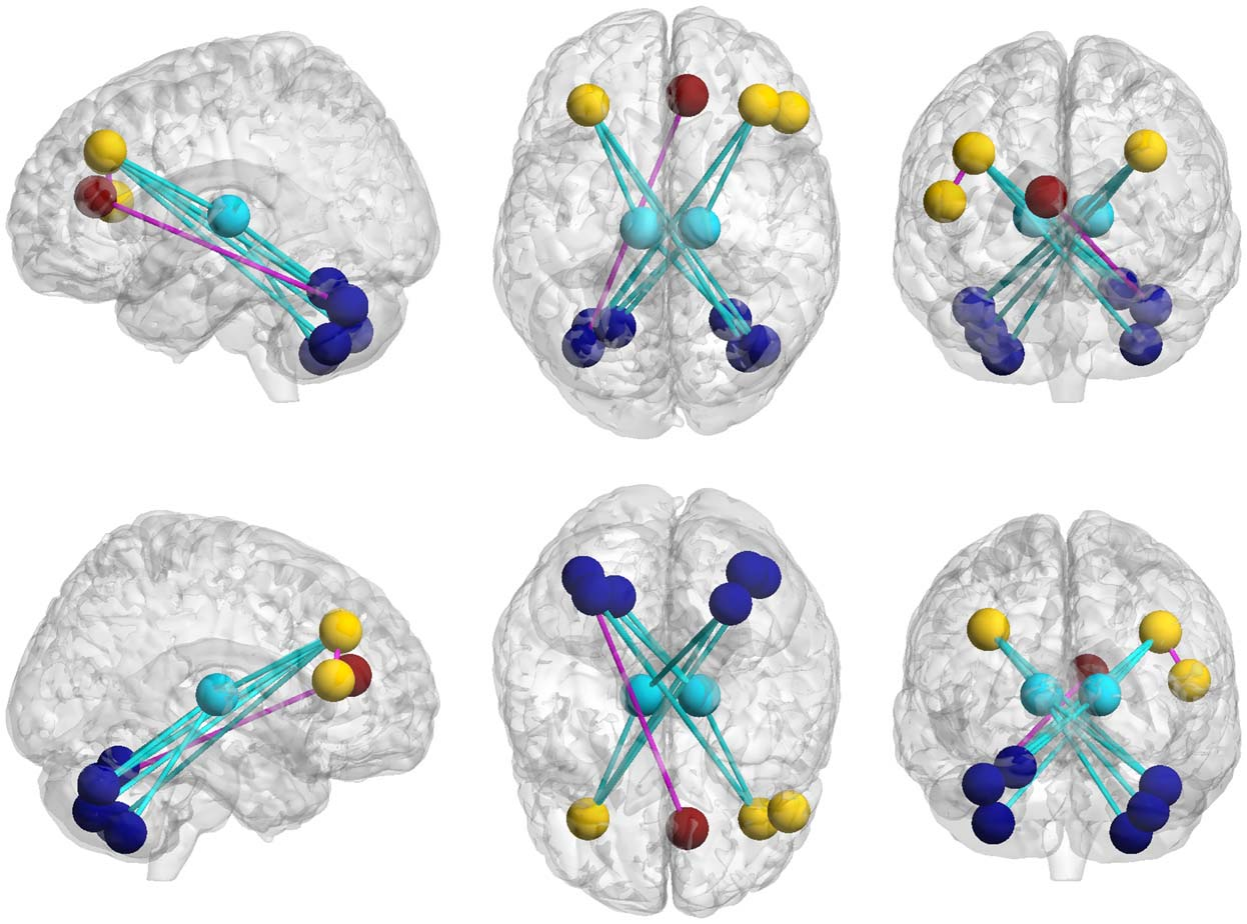
Differences in functional connectivity. Edges of each brain region (nodes) with indicated corresponding regions of decreased (blue) and increased (pink) functional connectivity in the PNE group.

**Figure 3:**
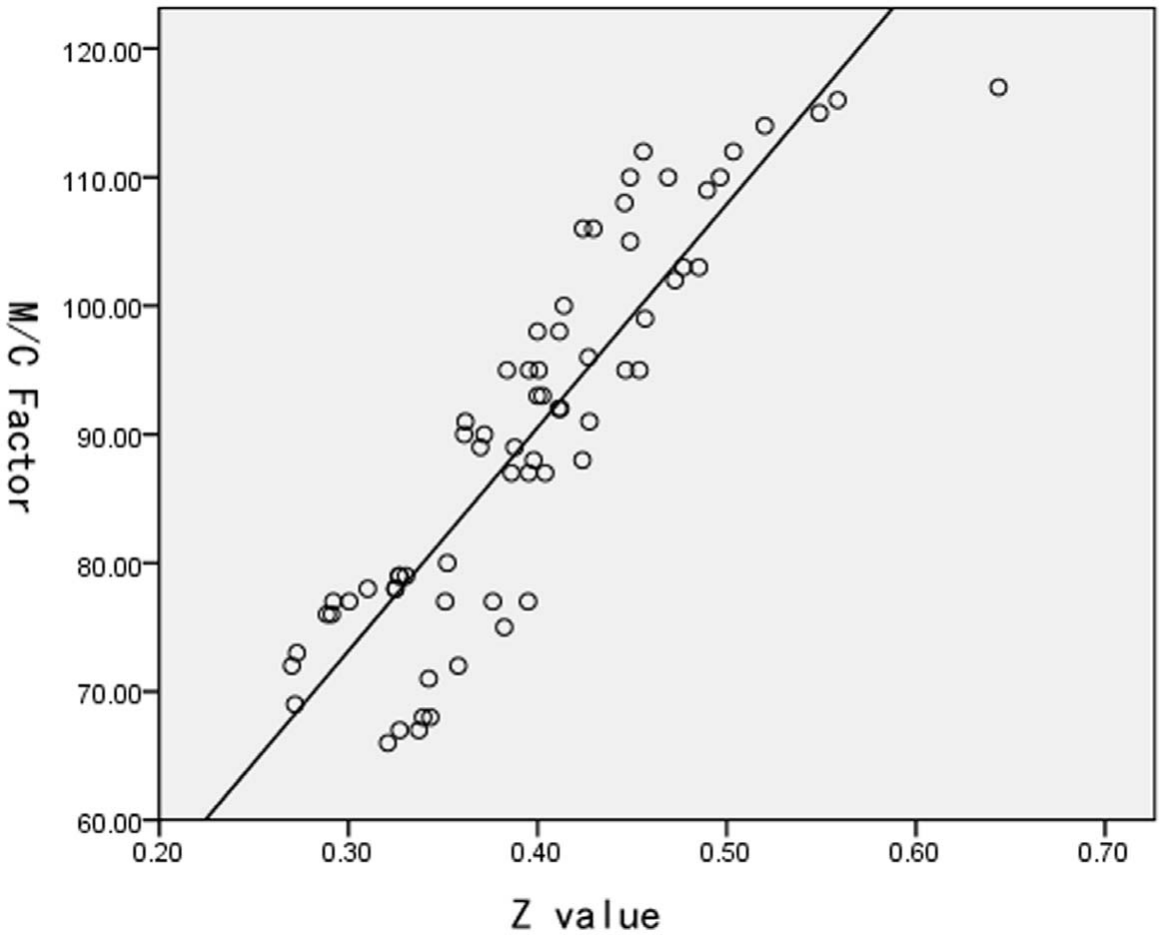
Relationship between M/C factor and connectivity z-scores. Relationship between M/C factor and connectivity z-scores between Cerebellum_Crus1_L and Frontal_Mid_R in PNE children (*r^2^* = 0.787, *P*<0.001).

Similar functional connectivity patterns were observed in both groups. The majority of strong functional connectivities (large z-scores) were observed between symmetrical interhemispheric regions, within a lobe, or adjacent to lobular regions (Fig. 1). A total of 12 significantly different functional connectivities were identified in the cerebellum, frontal lobe, and thalamus between the PNE and control groups at an FDR corrected threshold of *P*<0.05. We observed 10 decreased and 2 increased functional connectivities in the PNE group. Aberrant functional connectivities were visualized with the BrainNet Viewer (http://www.nitrc.org/projects/bnv/) (Fig. 2; Table 3). The M/C factor also showed a significant correlation with the z-scores representing connectivity between the Cerebellum_Crus1_L and Frontal_Mid_R (*r^2^* = 0.787, *P*<0.001) (Fig. 3).

**Table 3:** Differences in functional connectivity.

Connectivity		z-score		*P-*value_(corrected)_
		Control	PNE	
**Decreased**				
Frontal_Mid_R	Thalamus_R	0.3766	0.2449	3.1641 E-6
Frontal_Mid_L	Thalamus_L	0.3422	0.1659	3.9490 E-7
Cerebellum_6_L	Thalamus_R	0.7785	0.5717	3.5552 E-7
Cerebellum_Crus1_L	Thalamus_R	0.5671	0.3862	3.0981 E-5
Cerebellum_Crus1_R	Thalamus_L	0.3760	0.2669	7.9674 E-7
Cerebellum_8_R	Thalamus_L	0.2135	0.1576	4.1888 E-5
Cerebellum_Crus1_L	Frontal_Mid_R	0.5106	0.3351	1.0887 E-6
Cerebellum_7b_L	Frontal_Mid_R	0.5938	0.3740	6.9740 E-7
Cerebellum_Crus1_R	Frontal_Mid_L	0.5482	0.4149	5.8624 E-5
Cerebellum_Crus2_R	Frontal_Mid_L	0.6796	0.5317	1.3035 E-5
**Increased**				
Cingulum_Ant_R	Cerebellum_Crus1_L	0.1174	0.1905	4.3473 E-8
Frontal_Inf_Tri_R	Frontal_Mid_R	0.5203	0.6972	4.27437E-6

## Discussion

### C-WISC Testing Reveals Potential Attention Deficits in PNE

Intelligence testing was performed using the C-WISC method, which assesses parameters representative of speech, operation, and overall intelligence level. The current findings demonstrate that the overall intelligence level and verbal and manipulation abilities of PNE children were within normal ranges and were not significantly different from controls, indicating that PNE children have normal intelligence levels. Despite overall normal intelligence levels, children with PNE exhibited a markedly different M/C factor. The intelligence test revealed that PNE children had intelligence structure abnormality and attention deficits.

### Atypical Cerebello-thalamo-frontal Functional Connectivity in Children with PNE

The cerebello-thalamo-frontal pathway, including the left and right DLPFC, thalamus, and cerebellar hemisphere, were the predominant sites of altered functional connectivity. The cerebellum has been shown to have strong functional connectivities involved in working memory control and execution. Thus, this region has been associated with cerebellum dysfunctions related with attention deficit disorders in ADHD children using fMRI methodologies [24]–[27]. Our data suggest that aberrant connectivity of the cerebello-thalamo-frontal circuit may also be involved in the etiology underlying the initial development of attention deficit disorders in PNE children.

The thalamus is involved in normal attention functions; however, it also plays an important role in sleep cycle regulation and relaying sensory afferent information from the bladder. In the currently accepted model for bladder control during urine storage, this information is transmitted through the periaqueductal gray (PAG), to the ACC, the insula, and the lateral prefrontal cortex (LPFC) [28]. The current findings suggest that altered thalamic connectivity may result in an inability to wake during sleep in response to the need to void, and this is compounded by abnormally large urine storage volume in the bladders of children with PNE [29].

The ACC is involved in a wide range of cerebral functions, including cognitive function control, mediation of bodily arousal states, and interoceptive awareness [30]. The ACC is also involved in attention and introspection, which allows an individual to develop a sense of conscious awareness that the bladder is full. It also plays a role in executive control, a process involved in voiding the bladder in appropriate places and times [31]. The current study suggests that connectivity between the right ACC and left cerebellum (crus I) is increased in children with PNE, which may indicate functional changes in the cerebello-thalamo-frontal pathway that also affect other ACC functionalities. For example, the development of attention deficit disorders and nocturnal enuresis may involve similar mechanisms [11]. While the current study shows persuasive initial evidence for this hypothesis, the connectivities between the three distinguishable functional subregions of the ACC (dorsal, ventral, and circumcallosal) must be further examined in order to define the mechanistic role of the ACC in both PNE and attention deficit pathogeneses.

Children with PNE also exhibited a pattern of increased connectivity in the right inferior frontal gyrus. It has been suggested that neural reorganization may compensate for deficient regions during manipulation and memory maintenance in ADHD patients [32]. Thus, it follows that attention dysfunctions in children with PNE may also be correlated with abnormal cerebello-thalamo-frontal functional connectivity. The presence of attention deficit disorders would play a causative role in the failure to optimize the sense transduction pathways of the brain associated with bladder filling. Thus, inhibition of functional defects and subsequent induced functional disorder occur, ultimately resulting in PNE symptoms.

The functional connectivity abnormalities described here provide evidence initial to support these variations in children with PNE; however, the current study is limited by the relatively small, homogeneous sample. To confirm these results and their specific mechanism of action, future tests on large, varied cohorts will be required. The automated anatomical labeling atlas applied in the current study includes 116 regions, covering the entire cerebrum and cerebellum but excluding the brainstem. Thus, the current findings cannot rule out the involvement of this region in the pathogenesis of PNE; previous studies have suggested deficits in brainstem inhibition function in PNE children. Thus, brainstem functional connectivity should be investigated [3]. We should also note that subjects with PNE and ADHD were excluded to avoid the possible confounding effects of attention impairment caused by ADHD on experimental results. This exclusion may introduce certain elements of selection bias that must be considered when reviewing these results and designing future studies.

### Conclusions

We identified and attention deficit-based intelligence structure abnormality associated with variation in functional connectivities of the cerebello-thalamo-frontal region of the brain in children with PNE. This association between attention and PNE may provide valuable information to researchers interested in developing novel therapeutic treatments for persistent PNE in children and young adults.
